# Oligodendroglioma pseudoprogression after radiotherapy in a dog: a case report

**DOI:** 10.3389/fvets.2025.1572808

**Published:** 2025-05-09

**Authors:** Nicholas Rancilio, Mary Drozd, Logan Donaldson, Tyler Harm, Keiko Murakami

**Affiliations:** ^1^Department of Veterinary Clinical Sciences, Iowa State University, Ames, IA, United States; ^2^School of Veterinary Medicine and Biomedical Sciences, University of Nebraska-Lincoln, Lincoln, NE, United States; ^3^VCA Midwest Veterinary Emergency & Referral Center, Neurology & Neurosurgery, Omaha, NE, United States; ^4^Department of Veterinary Pathology, Iowa State University, Ames, IA, United States

**Keywords:** glioma, oncology, MRI, pseudoprogression, oligodendroglioma, brachycephalic, trigeminal nerve, cranial nerves

## Abstract

Pseudoprogression is a clinical and imaging phenomenon characterized by an increase in the size and contrast enhancement pattern of a glioma lesion following treatment with radiotherapy. In human beings, a substantial body of literature describes the phenomenon of pseudoprogression in glioblastoma after radiotherapy. The occurrence of gliomas in the cranial nerves has been reported in human beings as a clinically rare entity. A 7-year-old spayed female French Bulldog was presented with left-sided craniofacial muscle atrophy for a duration of 3 months and episodes of compulsive circling to the left. After a neurological examination, a magnetic resonance (MR) imaging scan of the brain was performed. A T2-and T2 FLAIR-weighted hyperintense, non-contrast-enhancing, T1-weighted hypointense intra-axial suprasellar lesion was found. In addition, an extra-axial, T1-weighted hyperintense, contrast-enhancing mass was identified at the level of the left trigeminal nerve. The lesions were presumptively diagnosed as a glioma and a left trigeminal nerve sheath tumor based on their imaging characteristics and the breed of the patient. A course of stereotactic radiotherapy (SRT) was prescribed, and 3 months after treatment, there was significant progression in the size of the suprasellar mass, indicative of either true progression or pseudoprogression. The left trigeminal nerve mass remained stable in size. Treatment with glucocorticoids resulted in a reduction in the size of the suprasellar mass, as observed on MR imaging 7 months after treatment. The left trigeminal nerve mass remained stable in size. Progression in the size of the suprasellar mass and the left trigeminal nerve mass occurred 9 months after the first course of treatment, and a second course of stereotactic radiotherapy was administered. Sixteen months after the first course of radiotherapy, a necropsy was performed. The suprasellar lesion and the left trigeminal nerve lesion were diagnosed as oligodendrogliomas on histopathology. Trigeminal nerve oligodendrogliomas and pseudoprogression following radiotherapy have not been previously described in dogs. Pseudoprogression should be considered a differential diagnosis for the progression of presumed or confirmed glioma lesions after treatment with radiotherapy. Concurrent oligodendroglioma lesions in the trigeminal nerve are also possible and should be included in the list of differential diagnoses for dogs with concurrent brain lesions.

## 1 Introduction

Pseudoprogression is a clinical and imaging phenomenon in gliomas, where the size and contrast enhancement pattern of the lesion on magnetic resonance (MR) imaging increases within 1 to 6 months following treatment with radiotherapy. The incidence of pseudoprogression varies widely, ranging from 9 to 30% in humans with gliomas (1–4). Pseudoprogression is most likely induced by a pronounced local tissue reaction involving inflammation, edema, and abnormal vessel permeability, leading to new or increased contrast enhancement on MR imaging (5, 6). Some studies have found an association between the incidence of pseudoprogression and improved survival, potentially attributable to an active inflammatory response against the tumor (7).

A decline in neurological function may accompany the imaging changes observed in pseudoprogression, or these imaging changes may be clinically silent. Pseudoprogression is managed conservatively with supportive care, such as glucocorticoids, or, in severe cases, with hospitalization and medical management of clinical signs (seizure medications, hypertonic saline, and mannitol). Serial imaging demonstrates a reduction in the lesion following the initiation of supportive care. Pseudoprogression of gliomas is well described in humans and presents a diagnostic challenge for neuro-oncologists in terms of appropriate case management (8). In this report, we present the case of a dog affected with a suprasellar oligodendroglioma and a concurrent trigeminal nerve oligodendroglioma, which was treated with stereotactic radiotherapy. The dog developed pseudoprogression 3 months after completing treatment, as evidenced by serial MR imaging at regular intervals. A summary timeline of key clinical events in this case is presented in Table 1. Understanding the phenomenon of pseudoprogression is important for confirming that post-treatment gliomas in dogs may behave similarly to those in human beings. In addition, clinicians managing dogs affected with gliomas should consider pseudoprogression following radiotherapy, as it can mimic the clinical signs of true tumor progression. Oligodendroglioma lesions in the trigeminal nerve are also possible and should be included in the list of differential diagnoses for dogs with concurrent brain lesions.

**Table 1:** Description of suprasellar oligodendroglioma lesion progression and interventions during treatment.

Time point	Suprasellar lesion longest diameter	% lesion longest diameter increase/decrease	RECIST response	Radiotherapy prescription	Prednisone dosages
0 (pretreatment)	17 mm	-	-	SRT 7 Gy x 3 fractions	0.5 mg/kg/day
3 months	24 mm	41%	Progressive	-	0.14 mg/kg/day
7 months	18 mm	6%	Stable disease	-	0.14 mg/kg/day
9 months	20 mm	18%	Stable (clinically progressive)	SRT 8 Gy x 3 fractions	0.5 mg/kg/day

## 2 Manuscript formatting

### 2.1 Headings

#### 2.1.1 Case description

A 7-year-old spayed female French Bulldog was presented with left-sided masseter and temporalis muscle atrophy for a duration of 3 months and episodes of compulsive circling to the left. Diffuse atrophy of the muscles of mastication was present; palpation of individual muscle bellies was clinically impossible due to the degree of atrophy and the patient’s conformation at the time of the initial presentation. A right head tilt and right-sided facial paralysis were also noted on clinical examination. Absence of left-sided facial sensation and corneal sensation was not detected. Neuroanatomical localization was multifocal in nature, with clinical signs localizing to the left trigeminal nerve (motor and sensory branches), the right vestibular system, the right facial nerve, the midbrain (mesencephalon), and the pons (metencephalon). Magnetic resonance imaging of the brain was subsequently performed. All MR images were acquired using a high-field GE Excite HDX 1.5 T magnet (Milwaukee, WI), and intravenous contrast was administered using gadolinium 0.2 mL/kg (Clariscan, GE Healthcare, Marlborough, MA), unless otherwise noted. Lesion measurements on MR imaging were performed by a board-certified radiologist and measured in mm at the widest diameter from the rostral-caudal (RC), medial-lateral (ML), and dorsal-ventral (DV) axes in all studies performed.

**Figure 1:**
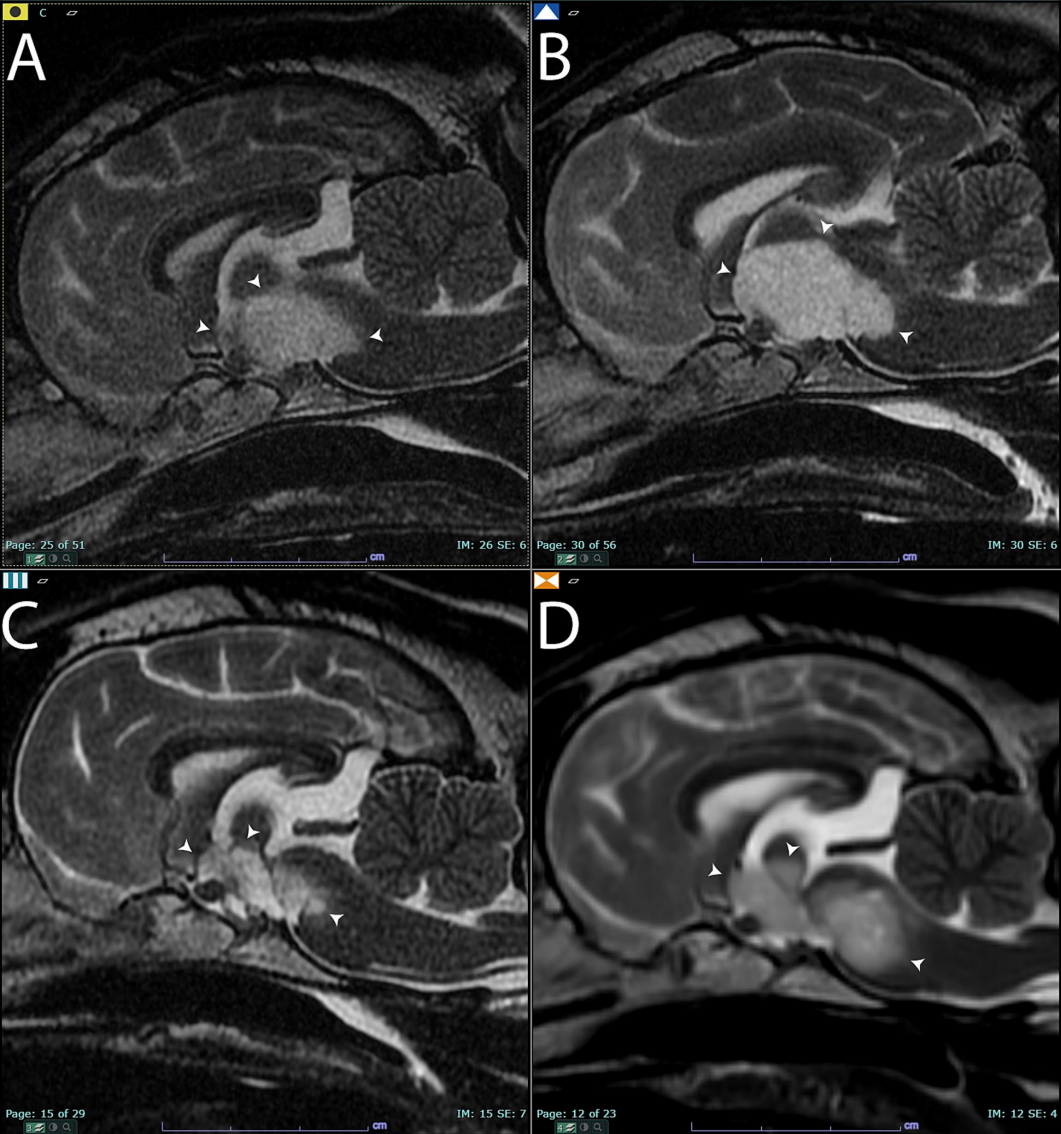
Sagittal T2-weighted images of the brain **(A)** before treatment. Arrowheads point to the T2 hyperintense lesion that was later diagnosed on histopathology as an oligodendroglioma. **(B)** Sagittal T2-weighted images of the brain 3 months after completing the first course of stereotactic radiotherapy. There was a qualitative and quantitative increase in the size of the mass (arrowheads) compared to panel **(A)**. Panel **(C)**, 7 months after completing stereotactic radiotherapy. The mass had significantly shrunk in size (arrowheads). Panel **(D)**, 9 months after completing stereotactic radiotherapy. Note the increased size of the mass (arrowheads) compared to panels **(A,C)**.

A T1-weighted hyperintense, extra-axial, contrast-enhancing mass was noted in the left trigeminal nerve at the level of the pons (metencephalon) (Supplementary Figures 1A, 6A). The lesion was T2-weighted and T2-weighted FLAIR hyperintense and was 7.5 mm at the widest diameter. A presumptive diagnosis of trigeminal nerve sheath tumor was made based on these imaging findings and the neurologic examination. A T1-weighted hypointense, non-contrast-enhancing intra-axial suprasellar lesion was also evident. This lesion was T2-weighted hyperintense and had a heterogenous pattern of T2-weighted FLAIR hypointensity and hyperintensity within and surrounding the lesion. The suprasellar lesion measured 17 mm x 12 mm x 14 mm (Figure 1A and Supplementary Figures 2A–D). Given the breed and imaging changes, this lesion was presumptively diagnosed as a glioma.

Computed tomography (CT) simulation was performed for radiotherapy treatment planning of the brain, and CT images of the abdomen were also acquired for staging purposes, in accordance with our institutional protocol for radiotherapy patients (Toshiba Aquillon LB 32 Slice, Canon, Tustin, CA). Computed tomography was performed with a 1-mm slice thickness, both prior to and following intravenous contrast administration (Omnipaque 240 mg/mL, 1 mL/lb., GE Healthcare, Chicago, IL). No planning target volume (PTV) was applied due to the size and proximity of each lesion to one another, as well as the sensitive location of the masses adjacent to the brainstem. For the suprasellar lesion, the gross tumor volume (GTV) was defined as all areas of abnormal T2-weighted FLAIR hyper/hypointensity and all areas of abnormal T1-weighted hypointensity. As the suprasellar lesion was poorly defined on CT imaging, CT was not used for lesion contouring. For the left trigeminal nerve mass, the GTV was defined as all abnormal T1 contrast-enhancing areas around the lesion. Any abnormal contrast-enhancing areas evident on CT imaging were also used to contour/define the GTV of the left trigeminal nerve mass. Stereotactic radiotherapy (SRT) was administered on consecutive weekdays using a linear accelerator, with 7 Gy × 3 fractions for a total dose of 21 Gy to both lesions. Treatment plans were developed using the Varian Eclipse treatment planning system, employing nine coplanar fixed gantry angle intensity-modulated radiotherapy fields (IMRT). The plan underwent a quality assurance check prior to delivery using the Varian portal dosimetry system and was calculated with the Varian Eclipse AAA v13.6 algorithm, utilizing a grid size of 1 mm. Prior to each fraction, a cone beam computed tomography (CBCT) scan was performed to verify patient positioning. All equipment used for administering and planning radiotherapy was manufactured by Varian (Clinac Ix Linear Accelerator with 6MV photons, Eclipse, Portal Dosimetry-Varian Medical Systems, Palo Alto, CA). The patient was also prescribed prednisone (0.5 mg/kg once daily) by mouth to mitigate any potential intracranial inflammation that could result from radiotherapy (PrednisTab, Lloyd, Shenandoah, IA, USA). Table 1 provides additional details about the prednisone dosages throughout the management of this case.

Three months after completing SRT, the patient was presented for re-evaluation and follow-up MR imaging. The patient continued to exhibit intermittent circling behavior, and the masticatory muscle atrophy on the left side remained unchanged from previous assessments. In the MR imaging, the lesion in the left trigeminal nerve was similar in size, shape, and T1-weighted post-contrast hyperintensity (Supplementary Figures 1B, 6B). However, the suprasellar lesion increased in size, measuring 24 mm x 15 mm x 10 mm at the widest diameters. The lesion appeared more T1-weighted hypointense than in the previous set of images, and a focal area of contrast enhancement was evident (Figure 1B and Supplementary Figure 3). The imaging changes were interpreted as either true progression of the presumed glioma or pseudoprogression, based on the response evaluation criteria in solid tumors v1.0 (RECIST) for dogs (9). The patient was continued on prednisone therapy but at a reduced dose due to reported clinical signs of anxiousness and panting at night (Table 1).

Six months after completing the first course of radiotherapy, the patient was presented with a ruptured corneal ulcer in the left eye. Enucleation and exenteration were performed, and the left globe was submitted for histopathology. No neoplasia was noted in the histopathologic examination of the left globe, and the diagnosis was characterized as perforating and ulcerative keratitis with corneal rupture and iris prolapse. Subacute fibrinosuppurative endophthalmitis with hyphema was also noted.

Seven months after the completion of the SRT course 1, the patient was re-evaluated. The circling behavior had resolved, and the MR imaging was repeated. The suprasellar mass lesion had decreased in size compared to previous imaging studies and measured 18 mm x 10 mm x 10 mm at the widest diameter. There was no contrast enhancement of the suprasellar mass, and it continued to have a similar pattern of heterogenous T2-weighted and T2-weighted FLAIR hyperintensity and hypointensity (Figure 1C and Supplementary Figure 4). Based on the RECIST criteria, the suprasellar mass was characterized as a stable disease compared to the pretreatment imaging. The left trigeminal nerve lesion remained unchanged in size and was also characterized as a stable disease based on the RECIST criteria at this same time point (Supplementary Figures 1C, 6C).

Nine months after the completion of SRT, the patient returned for evaluation due to the development of a right-sided head tilt and circling behaviors to both the left and right sides. A follow-up MR imaging study was performed using a 1.5 T high field magnet (GE Signa, Milwaukee, WI) both prior to and following the administration of 0.2 mL/kg of gadolinium (Clariscan, GE Healthcare, Marlborough, MA). Both the left trigeminal nerve (Supplementary Figures 1D, 6D) and suprasellar lesions were characterized as progressive disease based on the worsening clinical signs and an increase in size of the masses (Figure 1D and Supplementary Figure 5). The suprasellar lesion exhibited similar heterogenous T2-weighted and T2-weighted FLAIR hyperintensity and hypointensity, measuring 20 mm x 9 mm x 12 mm. Based on the clinical and imaging changes, a second course of SRT was prescribed using the same technique and equipment described above. A radiation prescription of 8 Gy x 3 fractions on consecutive weekdays was administered to both lesions, and the dose of prednisone was increased to 0.5 mg/kg per day by mouth. The increased prednisone dosage was chosen to limit the potential for radiotherapy-induced intracranial inflammation. The patient was euthanized 6 months after the second course of SRT due to the declining quality of life and worsening neurological clinical signs, as reported by the dog’s owner. Additional in-clinic follow-up was not performed after the completion of the second course of radiotherapy. The overall survival time was 16 months following the initiation of the first course of SRT.

#### 2.1.2 Necropsy findings

**Figure 2:**
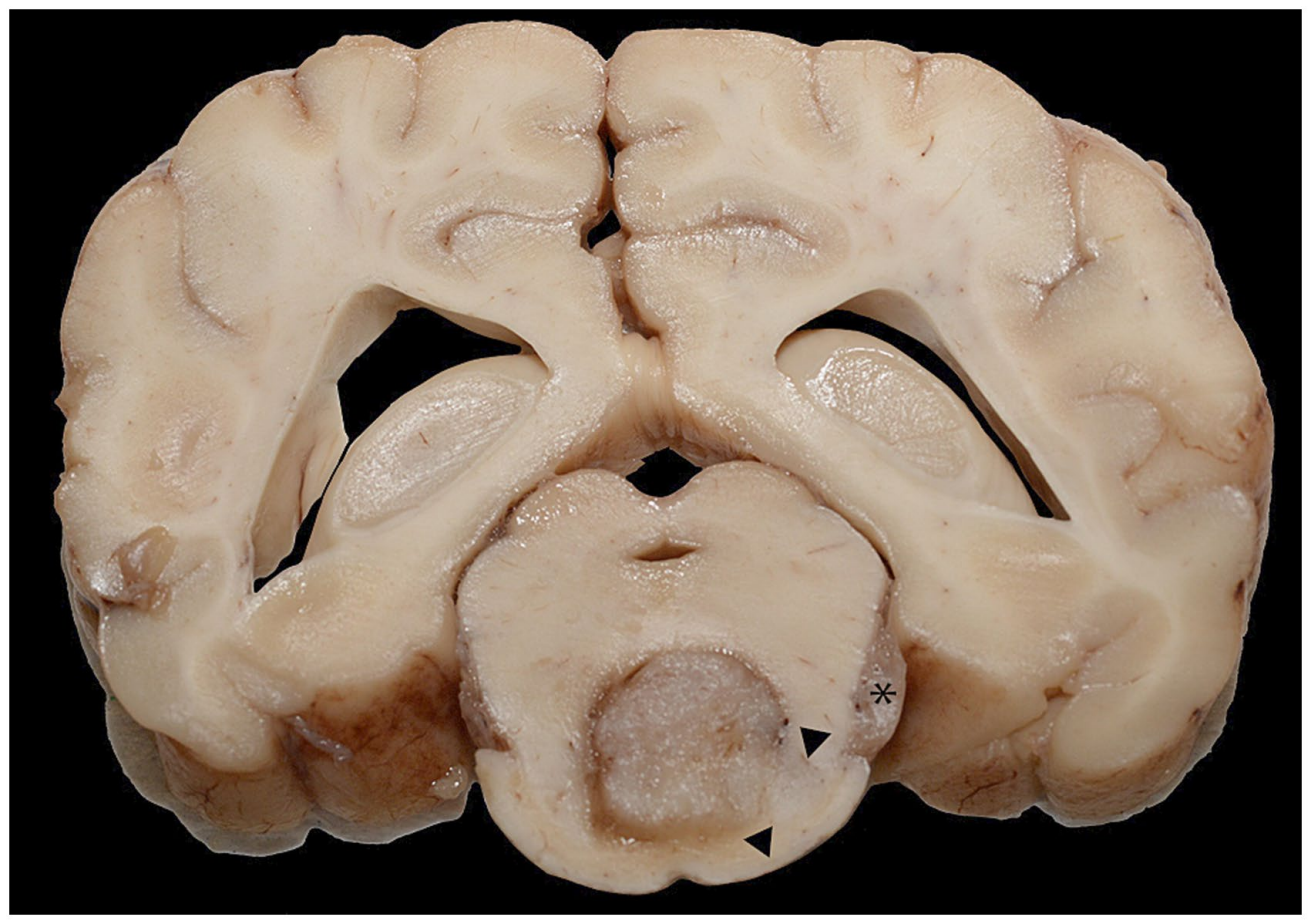
A cross-section of the brain at the level of the mid-brain formalin-fixed section. The ventral midbrain was effaced by the neoplastic mass, with pallor and infiltration of the neoplasm toward the ventral right margin and right of the central mass (arrowheads). There was a similar mass in the left trigeminal nerve (asterisk).

The left facial muscles were diffusely and markedly atrophied, and the left eye was absent. The left cranial nerves, including the maxillary and ciliary nerves, had ill-defined axonal margins compared to the contralateral nerves. The left ventral piriform lobe was enlarged and asymmetric, protruding (approximately 2 cm x 1 cm x 0.5 cm) along the ventral, medial, and rostral margins. The left half of the pons was similarly, mildly enlarged, and the adjacent meninges were dilated with watery, red fluid. On transection, the optic chiasm, fornix column, thalamus, third ventricle, and surrounding structures were effaced by necrosis. Proceeding caudally, transverse sections of the hypothalamus and ventral thalamus were severely to markedly effaced by a 9 mm x 6 mm, dark red to dark, indistinctly delineated neoplastic mass. The mass extended through successive sections of the mesencephalon and pons and infiltrated laterally toward the cerebellar peduncles (rostral and mid-cerebral peduncles). The areas of malacia extended through the center of the brain stem. The asymmetry of the gyri and sulci was occasionally associated with ill-defined areas of darker, softer neuropil (Figure 2).

Additional necropsy findings included acute, hemorrhagic typhlocolitis, lipid hepatopathy, and dermal hyperpigmentation and alopecia consistent with chronic corticosteroid treatment.

#### 2.1.3 Histopathologic descriptions

**Figure 3:**
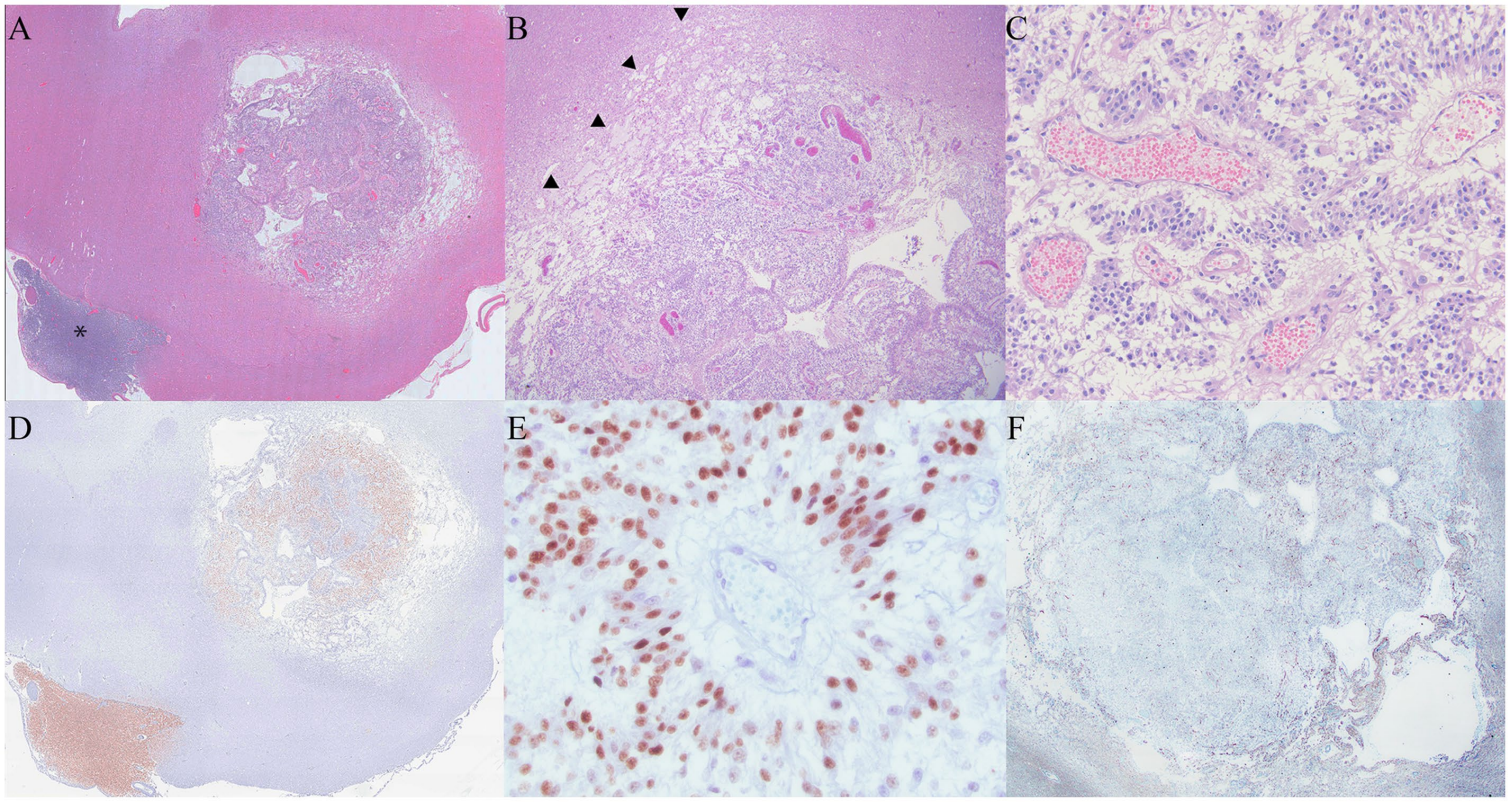
**(A)** At 1.5x magnification, the primary mass had a rim of pallor surrounding the neoplasm. The metastatic mass associated with the trigeminal nerve is identified with an asterisk. **(B)**: The rim of pallor (arrowheads) was composed of edema and necrosis consistent with pseudoprogression. **(C)**: Inset of 3c. The neoplasm was composed of polygonal cells arranged in the form of sheets and cords, infiltrated by tortuous, congested vessels. Pseudorossettes organized around congested vessels are features supportive of an oligodendroglioma diagnosis. **(D)** Neoplastic cells in both the primary mass and the mass overlying the trigeminal nerve were positive for Olig2 (Olig 2 immunohistochemistry). **(E)** At 400x magnification, Olig2 histochemistry staining was moderately to strongly positive within the neoplastic cells. **(F)** GFAP staining was negative within the primary neoplasm, and there was mild to moderate staining in the surrounding areas of necrosis.

Infiltrating the cranial nerves and ventral meninges and effacing the normal architecture of the hypothalamus and thalamus, the neoplastic mass extended into the brainstem and meninges of the cervical spinal cord. It was composed of polygonal cells arranged in sheets, cords, and pseudorosettes, interspersed with small, occasionally tortuous vessels. The surrounding neuropil was extensively effaced by abundant edema and mild to moderate glial inflammation, with occasional neuron degeneration. The neoplastic cells exhibited variable amounts of pale, eosinophilic to clear cytoplasm, with typically distinct cell borders. The irregular, round nuclei had coarse to stippled chromatin and occasionally contained a small nucleolus. There was mild anisocytosis, anisokaryosis, and pleomorphic variation. A total of 16 mitotic figures were counted in 2.4 mm^2^. The surrounding neuropil was extensively effaced by abundant edema, with mild to moderate glial inflammation and occasional neuron degeneration present. The neoplastic cells in the left trigeminal nerve and ganglion were similar to those in the primary mass (Figures 3A–C).

#### 2.1.4 Immunohistochemical findings

The neoplastic cells in multiple areas of the neoplasm were moderately to strongly positive for Olig2 and consistent with oligodendroglioma. Oligo2 highlighted areas of neoplastic cell infiltration into the surrounding neuropil. While GFAP was negative within the neoplasm, the areas of neoplasm infiltration overlapped with glial inflammation at the neoplasm margins (Figures 3D,E) (10).

## 3 Discussion

To the best of our knowledge, this is the first report of a dog with histological confirmation of malignancy describing pseudoprogression of an oligodendroglioma, with serial follow-up imaging after stereotactic radiotherapy. In a series of dogs treated with radiotherapy for presumed gliomas, one dog was reported to have clinically silent lesion progression 620 days after the completion of radiotherapy (11). The authors in this series speculated that this silent lesion progression might be pseudoprogression, but no necropsy or other histological confirmation of malignancy was performed. Pseudoprogression and late radiation necrosis have overlapping pathological and imaging characteristics, and there is controversy regarding the temporality of these phenomena (6). Late radiotherapy-induced brain necrosis does not occur until 6 months or later after the completion of treatment; pseudoprogression has also been reported in some cases 6 months after treatment (12). Therefore, the overlap in temporality between late radiation necrosis and pseudoprogression can confound clinicopathologic categorization when a progressive lesion occurs at the 6-month time point. In our case, the presence of a progressive lesion within 3 months of treatment, followed by regression of the lesion on serial imaging, places the observed findings within the category of pseudoprogression.

Canine brain tumors are reported to have a molecular overlap with human pediatric brain tumors (13). Pseudoprogression after radiotherapy or chemoradiotherapy is reported at higher rates in humans with MGMT promoter methylation in glioblastoma multiforme (14). The present case involves a high-grade oligodendroglioma, not glioblastoma multiforme. MGMT methylation is not a commonly reported characteristic of human oligodendroglioma. However, in human beings, a common driving mutation in oligodendroglioma development is IDH-1 mutations, which may also be present occasionally in canine oligodendroglioma (13). Pseudoprogression in human oligodendroglioma is also reported to occur in both IDH-1 wild-type and mutant oligodendrogliomas (15). The IDH-1 mutation status of our patient is unknown since testing for this mutation is not widely available in dogs.

This case presented a clinical challenge in determining recommendations for the owners regarding the next steps for continued management, as progressive lesions were found on serial MR imaging 3 months after the initial treatment. In our clinical experience, owners of brain tumor dogs with a progressive mass lesion on follow-up imaging often elect euthanasia due to the presumed lack of clinical response to treatment. However, the patient in the present case exhibited mostly mild clinical signs that were manageable on an outpatient basis, and euthanasia was discouraged. The finding of a progressive primary lesion and the likely diagnosis of a glioma required careful counseling of the owner regarding the need for serial follow-up imaging to characterize the behavior of the lesions. Therefore, this case highlights the importance of considering pseudoprogression in the differential diagnosis for dogs with a confirmed or strongly presumed glioma when progressive lesions are observed on the MR imaging after the completion of radiotherapy. Notably, despite the presence of pseudoprogression and true progression, the survival time in our patient is consistent with the survival time reported in the literature for presumed intracranial gliomas (11–21 months) (16–21).

While the patient in this report was treated with prednisone throughout the course of the disease, the inclusion of this medication is unlikely to have had much effect on the outcome. The dog was on the highest dose of prednisone before developing the pseudoprogressive lesion and was subsequently tapered to lower doses of prednisone when pseudoprogression was noted due to reported anxiousness and panting. Prednisone was initiated before radiotherapy with the intention of gradually reducing the dose to the lowest effective dose for controlling neurologic clinical signs. Until rather recently, prednisone dosing in veterinary radiotherapy for brain tumors has been anecdotal and not based on high levels of evidence. Prospective data that demonstrate the safety of rapidly tapering the dose of prednisone after the completion of radiotherapy for brain tumors have become available (22). The present case was treated prior to the publication of this data; therefore, the exact protocol from the aforementioned manuscript was not followed. Prednisone dosage was increased before starting a second course of radiotherapy, which was consistent with the findings of our clinical experience.

During treatment, the patient developed clinical signs consistent with a left corneal ulcer and rupture after the first course of radiotherapy. A subsequent neurological examination of this patient indicated no complete loss of corneal sensation or facial paralysis on the left side. It is possible that radiotherapy could have damaged the facial nerve due to the proximity of the treatment to the facial canal, and the presence of these clinical findings could indicate a late side effect of radiotherapy. No evidence of tumor extension into the left globe was found upon histological examination of the eye after enucleation.

The patient in the present case had a concurrent oligodendroglioma lesion in the left trigeminal nerve and ganglion. Trigeminal nerve oligodendroglioma has not been reported in dogs, and there are very few reports of its occurrence in human beings, with a total of 16 cases (23, 24). It is speculated that the extension of primary oligodendroglioma into the trigeminal nerve may occur through the infiltration of the tumor along the white matter tracts and through the root entry zone and proximal cisternal segments (23, 24). It is also well documented that oligodendrogliomas in dogs are capable of drop metastasis throughout the central nervous system. Drop metastasis is believed to occur when tumor cells seed through the ventricular system, leading to metastases distant from the primary tumor, appearing throughout the rest of the brain and spinal cord as the disease progresses (25–27). It is unknown in the present case whether the concurrent oligodendroglioma lesions represent drop metastasis, extension of one of the lesions to the other location, or simply concurrent primary tumors (independent events). We were unable to demonstrate antemortem or postmortem evidence for any of these scenarios.

In conclusion, pseudoprogression of oligodendrogliomas occurs in dogs and should be considered in the differential diagnoses of progressive masses and clinical signs following the completion of radiotherapy. Concurrent lesions in the cranial nerves, such as the trigeminal nerve, may also be oligodendrogliomas and should be included in the list of differential diagnoses.

## Data Availability

The raw data supporting the conclusions of this article will be made available by the authors, without undue reservation.

## Supplementary material

SUPPLEMENTARY FIGURE 1 Axial T1 weighted +C images at the level of the rostral pons **(A–D)**. Note the contrast enhancing T1 hyperintense extra-axial lesion at the left trigeminal nerve **(A)**. This lesion was diagnosed as an oligodendroglioma. **(B)** 3 months after treatment with radiotherapy the oligodendroglioma remains stable in size. **(C)** 7 months after treatment with radiotherapy. (D) 9 months after treatment with radiotherapy the lesion has progressed in size. Note also the left tympanic bulla in figure **(D)** contains effusion. This is likely due to dysfunction of the eustachian tube secondary to denervation of the tensor veli palatini muscle which is innervated by the mandibular branch of the trigeminal nerve.

SUPPLEMENTARY FIGURE 2 Axial images at the level of the pituitary gland demonstrating the pre treatment appearance of the oligodendroglioma. **(A)** T1 weighted images, **(B)** T1 weighted+Contrast images, **(C)** T2 weighted images, **(D)** T2 FLAIR weighted images. The tumor is delineated by the arrows in each panel.

SUPPLEMENTARY FIGURE 3 Axial images at the level of the pituitary gland 3 months after treatment with radiotherapy demonstrating pseudoprogression of the oliogodendroglioma. **(A)** T1 weighted images, **(B)** T1+Contrast weighted images-note the area of new contrast enhancement (*), **(C)** T2 weighted images, **(D)** T2 FLAIR weighted images. The lesion is delineated by the arrows in each panel.

SUPPLEMENTARY FIGURE 4 Axial images at the level of the pituitary gland 7 months after treatment with radiotherapy demonstrating decrease in size of the oligodendroglioma lesion. **(A)** T1 weighted images, **(B)** T1 weighted +Contrast image, **(C)** T2 weighted images, **(D)** T2 FLAIR weighted images. The lesion is delineated by the arrows in each panel. The area of contrast enhancement around the lesion is no longer visible in panel **(B)** or any of the other planes examined.

SUPPLEMENTARY FIGURE 5 Axial images at the level of pituitary gland 9 months after treatment with radiotherapy demonstrating progression in size of the oligodendroglioma lesion. **(A)** T1 weighted images, **(B)** T1 weighted +Contrast image, **(C)** T2 weighted images, **(D)** T2 FLAIR weighted images. The lesion is delineated by the arrows in each panel.

SUPPLEMENTARY FIGURE 6 Dorsal T1 weighted + contrast images at approximately the level of the trigeminal nerve/trigeminal ganglion. **(A)** prior to treatment. **(B)** 3 months after treatment with radiotherapy. **(C)** 7 months after treatment with radiotherapy. **(D)** 9 months after treatment with radiotherapy. The arrow heads in each panel demonstrate the extra-axial lesion in the trigeminal nerve. It was not possible to image the trigeminal ganglion exactly at each time point due to differences in positioning, machines, and differences in size of the primary suprasellar lesion causing displacement of normal structures.
