# Supplementary figures and images for: Oligodendroglioma pseudoprogression after radiotherapy in a dog: a case report
Source: Front Vet Sci. 2025 May 9;12:1572808. doi: 10.3389/fvets.2025.1572808 (PMC12100751; doi:10.3389/fvets.2025.1572808)

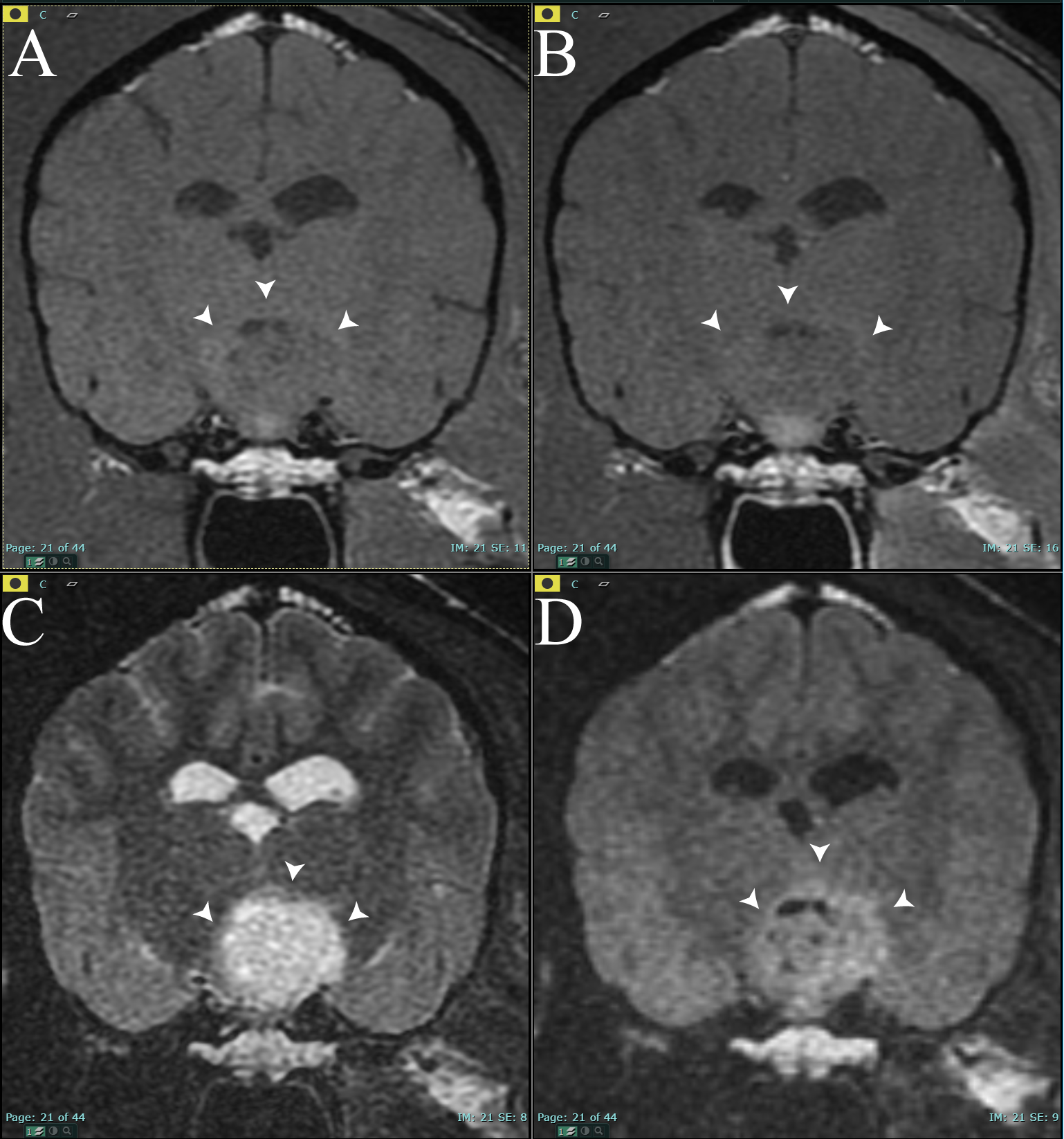

Supplement: SUPPLEMENTARY FIGURE 2 — Axial images at the level of the pituitary gland demonstrating the pre treatment appearance of the oligodendroglioma. (A) T1 weighted images, (B) T1 weighted+Contrast images, (C) T2 weighted images, (D) T2 FLAIR weighted images. The tumor is delineated by the arrows in each panel. [file Image_2.tif]

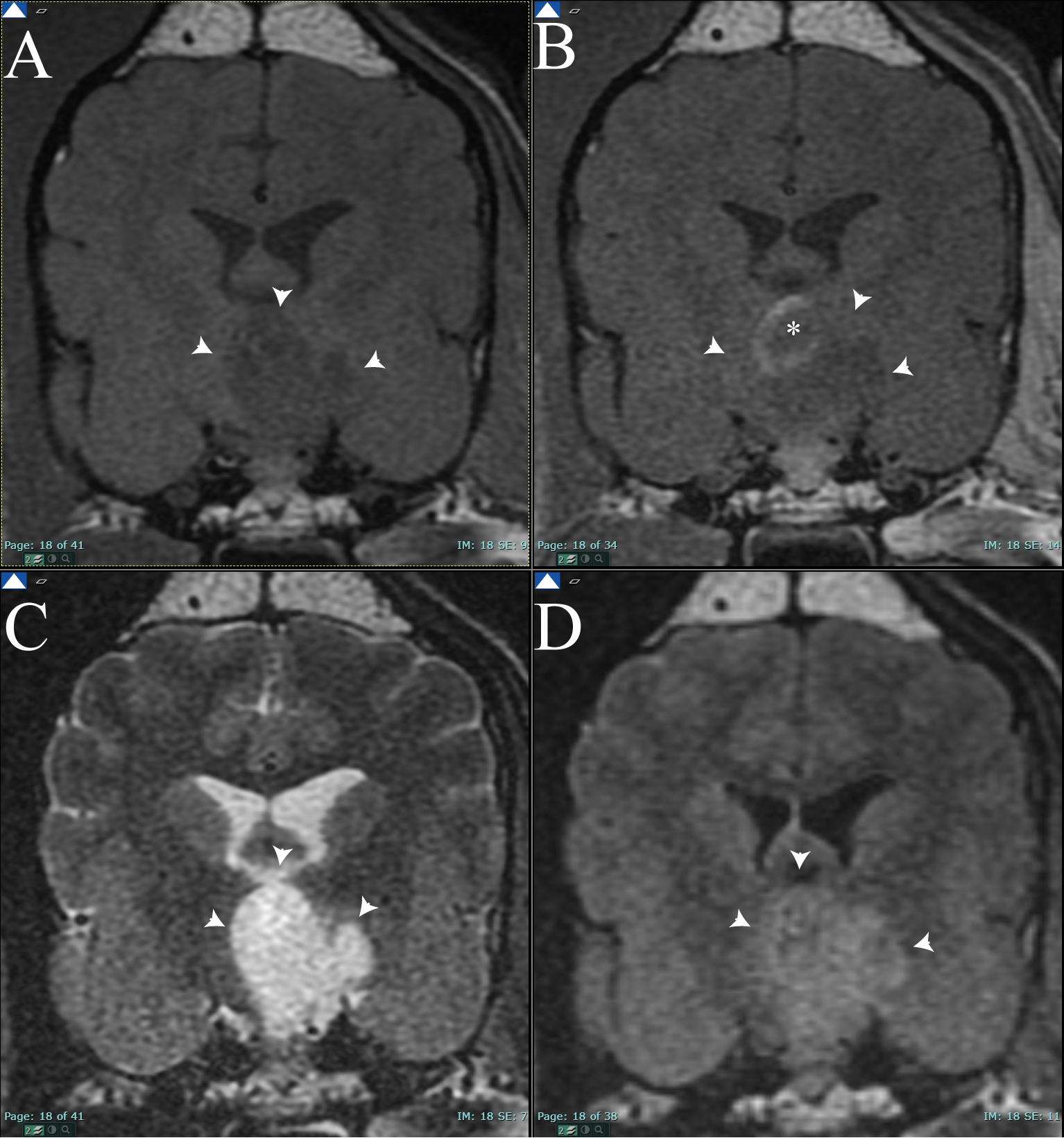

Supplement: SUPPLEMENTARY FIGURE 3 — Axial images at the level of the pituitary gland 3 months after treatment with radiotherapy demonstrating pseudoprogression of the oliogodendroglioma. (A) T1 weighted images, (B) T1+Contrast weighted images-note the area of new contrast enhancement (*), (C) T2 weighted images, (D) T2 FLAIR weighted images. The lesion is delineated by the arrows in each panel. [file Image_3.tif]

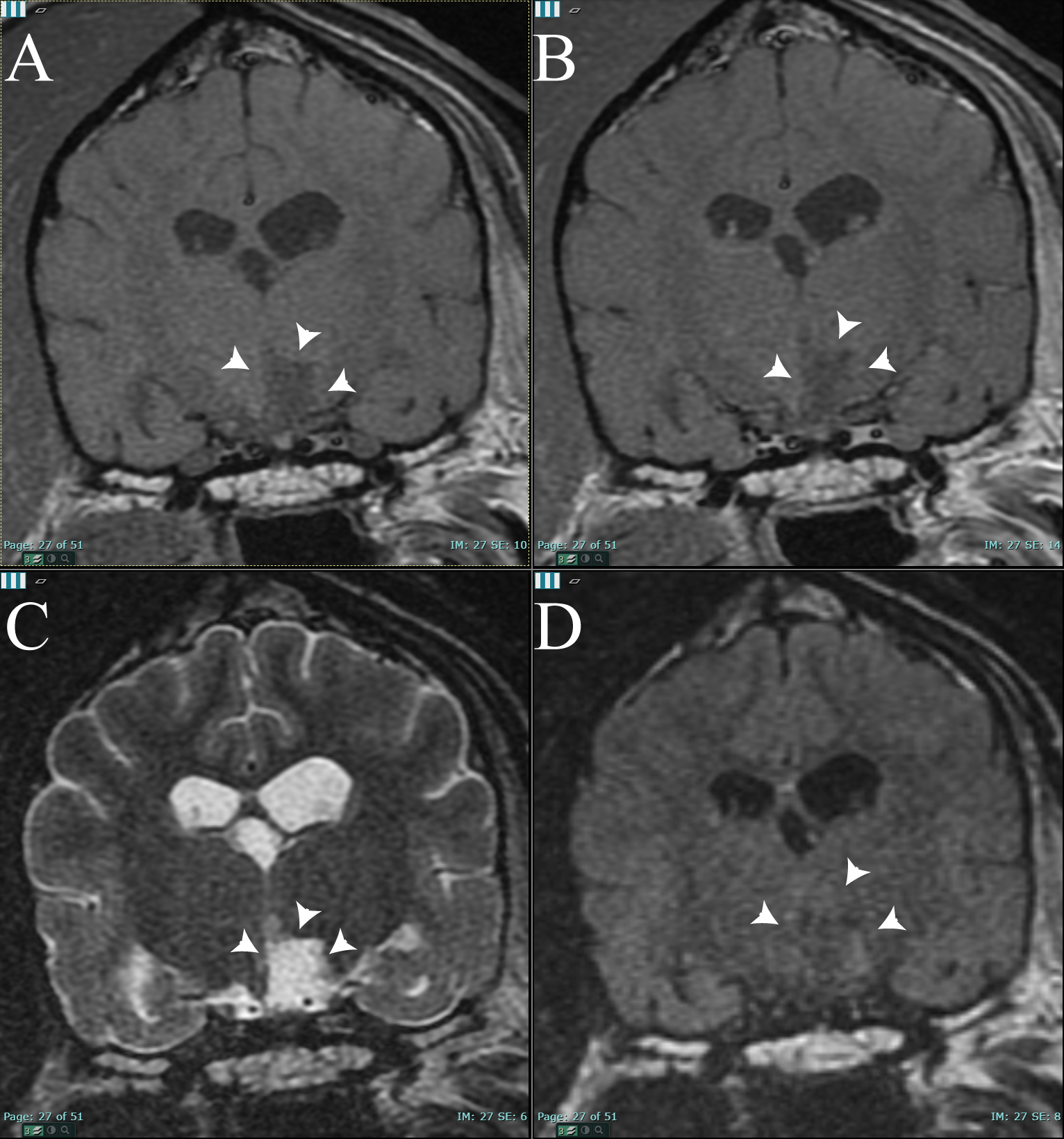

Supplement: SUPPLEMENTARY FIGURE 4 — Axial images at the level of the pituitary gland 7 months after treatment with radiotherapy demonstrating decrease in size of the oligodendroglioma lesion. (A) T1 weighted images, (B) T1 weighted +Contrast image, (C) T2 weighted images, (D) T2 FLAIR weighted images. The lesion is delineated by the arrows in each panel. The area of contrast enhancement around the lesion is no longer visible in panel (B) or any of the other planes examined. [file Image_4.tif]

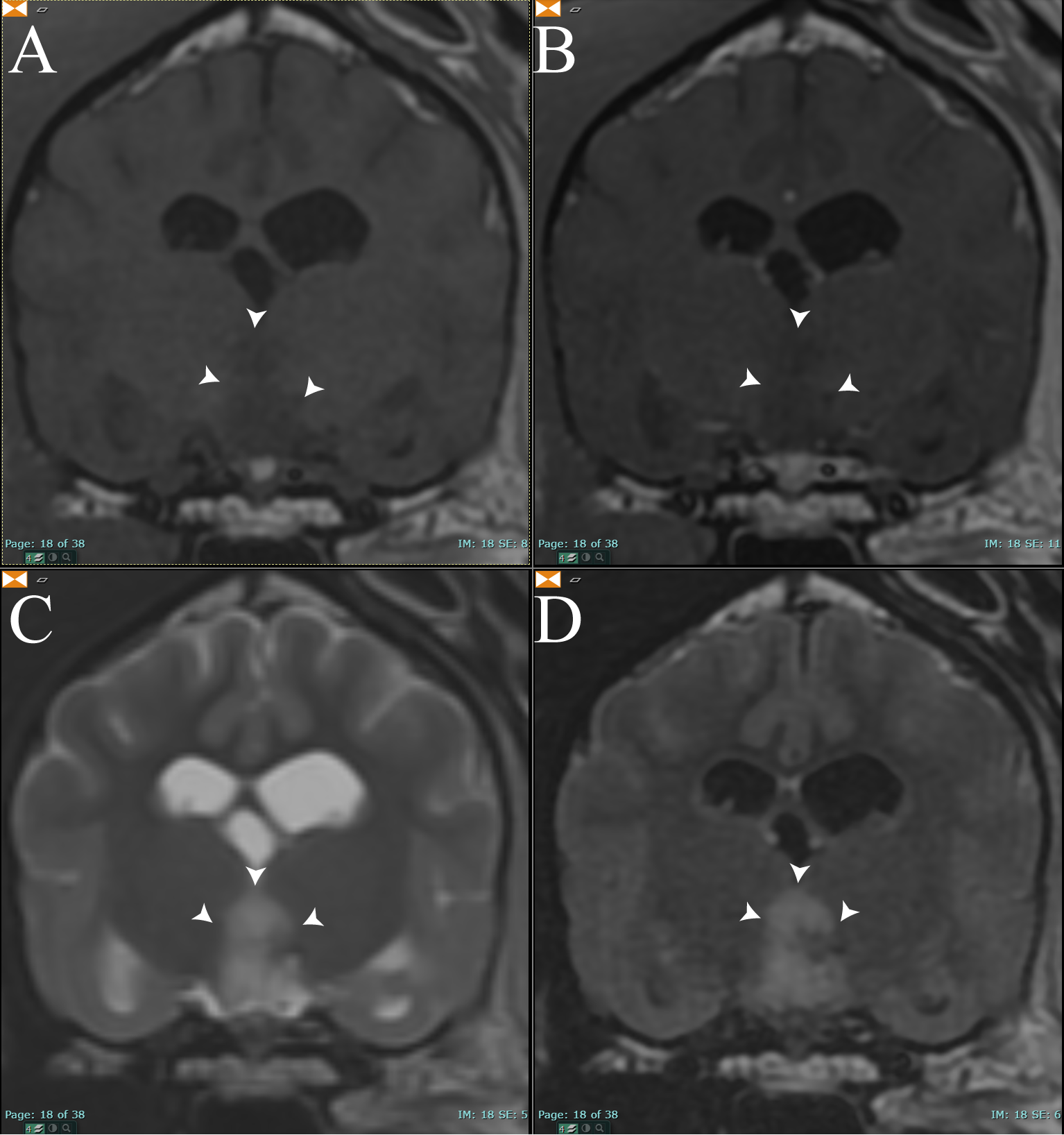

Supplement: SUPPLEMENTARY FIGURE 5 — Axial images at the level of pituitary gland 9 months after treatment with radiotherapy demonstrating progression in size of the oligodendroglioma lesion. (A) T1 weighted images, (B) T1 weighted +Contrast image, (C) T2 weighted images, (D) T2 FLAIR weighted images. The lesion is delineated by the arrows in each panel. [file Image_5.tif]
